# The Role of Cellular Coupling in the Spontaneous Generation of Electrical Activity in Uterine Tissue

**DOI:** 10.1371/journal.pone.0118443

**Published:** 2015-03-20

**Authors:** Jinshan Xu, Shakti N. Menon, Rajeev Singh, Nicolas B. Garnier, Sitabhra Sinha, Alain Pumir

**Affiliations:** 1 College of Computer Science, Zhejiang University of Technology, Hangzhou, China; 2 The Institute of Mathematical Sciences, CIT Campus, Taramani, Chennai 600113, India; 3 Laboratoire de Physique, Ecole Normale Supérieure de Lyon, F-69007, Lyon, France; 4 Max-Planck Institute for Dynamics and Self-Organisation, D-37073, Göttingen, Germany; National Scientific and Technical Research Council (CONICET)., ARGENTINA

## Abstract

The spontaneous emergence of contraction-inducing electrical activity in the uterus at the beginning of labor remains poorly understood, partly due to the seemingly contradictory observation that isolated uterine cells are not spontaneously active. It is known, however, that the expression of gap junctions increases dramatically in the approach to parturition, by more than one order of magnitude, which results in a significant increase in inter-cellular electrical coupling. In this paper, we build upon previous studies of the activity of electrically excitable smooth muscle cells (myocytes) and investigate the mechanism through which the coupling of these cells to electrically passive cells results in the generation of spontaneous activity in the uterus. Using a recently developed, realistic model of uterine muscle cell dynamics, we investigate a system consisting of a myocyte coupled to passive cells. We then extend our analysis to a simple two-dimensional lattice model of the tissue, with each myocyte being coupled to its neighbors, as well as to a random number of passive cells. We observe that different dynamical regimes can be observed over a range of gap junction conductances: at low coupling strength, corresponding to values measured long before delivery, the activity is confined to cell clusters, while the activity for high coupling, compatible with values measured shortly before delivery, may spread across the entire tissue. Additionally, we find that the system supports the spontaneous generation of spiral wave activity. Our results are both qualitatively and quantitatively consistent with observations from *in vitro* experiments. In particular, we demonstrate that the increase in inter-cellular electrical coupling observed experimentally strongly facilitates the appearance of spontaneous action potentials that may eventually lead to parturition.

## Introduction

It is well known that the contraction of uterine smooth muscle cells (myocytes) is triggered by electrical activity resulting from action potentials that evolve from single spikes to spike trains in the lead up to parturition [1–3]. However, the precise mechanism underlying the transition of the uterus from the quiescent organ seen during most stages of pregnancy, to the rhythmically contracting muscle observed at the onset of labor, remains to be fully explained. Preterm births, which occur prior to 37 weeks of gestation, can spontaneously arise from early, undesired uterine contractions [4], and hence this process is highly significant from a clinical perspective. Indeed, recent data suggests that preterm births constitute approximately 10% of all births [5], and that rates of spontaneous preterm labor have been increasing at the same rate as elective or induced preterm births [6]. Preterm births have been implicated as the cause of over a million neonatal deaths per year worldwide, and in around 50% of all cases of infant neurological damage [4]. In the USA alone, they have been linked to 40% of all infant deaths [7]. A clearer understanding of the mechanism of spontaneous uterine tissue contraction could therefore greatly facilitate the development of effective strategies to help curb neonatal mortality and morbidity.

It has been postulated that spontaneous electrical oscillations in human uterine tissue, observed prior to the mechanical contractions of the pregnant uterus, may be initiated by “pacemaker” cells [8, 9], similar to the Interstitial cells of Cajal, which are known to act as a pacemaker in other smooth muscles, such as the rabbit urethra [10, 11]. However, despite much effort aimed at identifying the origin of spontaneous uterine contractions [12], there has thus far been no clear evidence for the existence of such cells in the human uterus. On the contrary, uterine tissue is known to contain an abundance of electrically passive cells, such as Interstitial Cajal-like Cells (ICLCs) or telocytes [13] which, despite their similarity to Cajal cells, have been argued to inhibit electrical activity [11, 14]. ICLCs have a density of about 100 – 150 cells/mm^2^ in the uterus, and contribute up to ∼18% of the cell population immediately below the mucosal epithelium [14, 15]. Their density is highest on the surface of the uterus and decreases to a value of around 7.5% in muscularis [14]. Other electrically passive uterine cells include fibroblasts [14], which play an important role in remodeling the human uterine cervix during pregnancy and parturition [16, 17].

The hypothesis that spontaneous electrical behavior is an inherent property of uterine smooth-muscle cells [8] has gained traction as it is known that numerous electrophysiological changes occur in the myometrium during the course of pregnancy. In human uterine tissue, the myocyte resting potential has been observed to change from a value close to −70*mV* at the beginning of pregnancy to around −55*mV* at midterm [18]. These changes can be related to the morphological modulations of the uterine tissue [3], that are particularly apparent shortly before delivery. As the tissue enlarges to accommodate the growing fetus, its weight increases from around 75g to 1300g in humans [19]. However, it has not yet been demonstrated that such changes are responsible for the spontaneous generation of action potentials necessary for the periodic mechanical contractions of the uterine tissue [19].

Studies on rats uterine tissue generally corroborate the picture obtained in the human uterus. In particular, the results of [20] do not reveal the presence of any pacemaker cells. The changes in the outward (K^+^) and inward (Na^+^, as well as Ca^2+^) currents have been documented [21, 22]. It has been observed from experiments on rat uterine myocytes that the recorded peak current in the Na^+^ channel increases from ∼ 2.8*μ*A/cm^2^ in a non-pregnant uterus to ∼ 5.1*μ*A/cm^2^ at late pregnancy, while the corresponding peak current in the Ca^2+^ channel decreases from ∼ 5.7*μ*A/cm^2^ to ∼ 3.4*μ*A/cm^2^ over the same range [21]. These changes are accompanied by changes in both the surface area of a single myocyte cell, from ∼ 1930*μ*m^2^ to ∼ 7600*μ*m^2^ during late pregnancy, and, consistent with the observed hypertrophy of the uterus, a five-fold increase in the cell capacitance [21].

In rat uterine tissue, an alternative paradigm for the genesis of coherent uterine activity is hinted at by the fact that an even more dramatic change occurs close to term in the uterus. The fractional area of gap junctions, defined as the ratio of the membrane area occupied by gap junctions to the total membrane area, has been observed to show a 20-fold increase in the rat uterus [23]. Furthermore, the gap junctional conductance has been found to increase from ∼ 4.7 nS at normal preterm to ∼ 32 nS during delivery [24], while a reduced expression of the major gap junction protein connexin 43 in transgenic mice is known to significantly delay parturition [25]. It is well-known that the electrical coupling by gap junctions affects the conduction velocity of waves of activity propagating through the tissue: propagation in cardiac tissue is only possible when the coupling is strong enough, and the velocity of propagation increases with the coupling strength, i.e., the gap junction expression [26]. The importance of gap junction expression is manifested most spectacularly in the observation that chemical disruption of the gap junctions immediately inhibits the oscillatory uterine contractions [27–29]. These findings strongly suggest that gap junctional coupling between proximate cells plays a very important role in the development of coordinated uterine electrophysiological activity, and may be responsible for the transition from the weak, desynchronized myometrial contractions seen in a quiescent uterus to the strong, synchronous contractions observed during labor [23, 24].

It has recently been observed that the coupling of an excitable cell to an electrically passive cell in a simple theoretical model of myocyte activity can give rise to oscillations, even if neither of the cells are initially oscillating [30]. This prediction seems to be borne out by experiments, as the coupling of electrically active and passive cells in an assembly is indeed known to significantly affect the observed synchronization dynamics [31, 32], while complicated dynamical regimes are observed in preparations of weakly coupled cardiac myocytes [33, 34]. The physiological significance of this phenomenon can be inferred from the fact that the activity synchronizes at high coupling strengths in both experimental preparations and numerical simulations of theoretical models. This synchronization first occurs over small regions (cell clusters) whose size gradually increases to fill out the full media, so that all cells beat with the same frequency [31, 35, 36].

Consequently, it has been hypothesized [36] that spontaneous oscillatory behavior could be initiated by the strong increase in coupling between non-oscillating electrically active and passive cells of a pregnant uterus shortly before delivery. Further justification for this claim stems from the fact that in rat uterine tissue, close contact between ICLCs and smooth muscle cells has been observed [20]. Moreover, while there has thus far been no direct evidence for the electrical coupling between myocytes and fibroblasts via gap junctions, analogous *in vitro* studies of rats [37] or rabbits [38] cardiac tissue strongly suggest the occurrence of such coupling. The resting potential of ICLCs, $V_{I}^{r}$ is around $V_{I}^{r}∼-58\pm 7mV$ [20] and, while the resting potential of fibroblasts, $V_{F}^{r}$, varies over a large range (−70 mV to 0 mV), it is mostly in the range $-25mV\leq V_{F}^{r}\leq 0mV$ (in 77.3% of all cases), with a peak of the distribution at −15*mV* [39]. As both cells have resting potentials larger than that of the myocyte, they can act as a source of depolarizing current on coupling, and are thus potentially significant participants in the generation of spontaneous activity [30, 36]. However, the argument that spontaneous uterine activity is a result of coupling between electrically active and passive cells has thus far been tested only on a highly simplified model of myocyte electrical activity [36], and no significant attempt has yet been made to relate the model parameters to actual observations.

Recently developed realistic, biologically detailed models of uterine myocytes [40, 41] allow for a precise theoretical study of the roles of individual physiological components in the generation of desirable, as well as pathological, electrical activity, which in turn permits a better understanding of their correlation with contractile force [42, 43]. The purpose of the present work is to investigate the effect of cell coupling on spontaneous electrically activity using an electrophysiologically realistic mathematical model, and to examine the synchronization behaviour that occurs when this coupling is sufficiently strong. To this end, we present a model for the electrical activity of uterine smooth muscle cells coupled to passive cells. This model is based on a realistic mathematical description of rat uterine smooth muscle cell electrical activity recently developed by Tong *et al*. [41], which uses a general Hodgkin-Huxley formalism to describe the evolution of the membrane potential of myocytes, *V*_*m*_, and the Calcium ionic concentration in the cytosol, [Ca^2+^]_i_. The details of our model are discussed in the Methods section. The most significant modification we make to the model of Tong *et al*. is the addition of an extra current, arising from the electrical coupling, and an associated equation for the evolution of the passive cell potential.

In the following section, we present the results of a systematic investigation into the conditions that give rise to spontaneous electrical activity, in particular the dependence of myocyte activity on gap junction conductivity, passive cell resting potential and the number of passive cells. Furthermore, we examine the regimes that arise when myocytes and passive cells are coupled in a two-dimensional (2-D) assembly. This 2-D configuration mimics a cell culture of the type routinely used in cardiac preparations [33, 34], or in experiments performed on the pregnant uteri of small animals [44], thus facilitating the potential experimental verification of our observations. Our numerical results strongly suggest that coupling plays an important role in both the appearance of oscillations as well as in the emergence of synchronized activity in the tissue. Moreover, we find that our model is capable of capturing rich dynamical regimes, characterized by periodically spaced, irregular patterns of action potentials, that are qualitatively consistent with recent observations [44].

## Materials and methods

Our mathematical model builds upon the description of rat uterine smooth muscle cell activity developed by Tong *et al*. [41], which consists of a set of first order ordinary differential equations that describe the evolution of fourteen ionic currents, including depolarizing Na^+^ and Ca^2+^ currents and repolarizing K^+^ currents. The description of each ionic current involves activating and inactivating gating variables, *m*_*h*_ which specify the state of each channel *h*, and are governed by evolution equations of the type:

$$\begin{array}{c} \frac{dm_{h}}{dt}=\frac{m_{h}^{\infty }-m_{h}}{\tau_{h}}\;, \end{array}$$ (1)

where $m_{h}^{\infty }(=\alpha_{h}/(\alpha_{h}+\beta_{h}))$ are the asymptotic values of *m*_*h*_, *τ*_*h*_(= 1/(*α*_*h*_ + *β*_*h*_)) are the relaxation times, and *α*_*h*_ (*β*_*h*_) are the rates at which the channels open (close). The relaxation times are represented by nontrivial functions of the membrane potential, *V*_*m*_, that are typically determined experimentally.

We describe the excitation dynamics of myocytes in terms of the time evolution of this membrane potential:

$$\begin{array}{c} C_{m}\frac{\text{d}V_{m}}{\text{d}t}=-I_{\text{ion}}+I_{\text{ext}}+I_{\text{gap}}\;, \end{array}$$ (2)

where *C*_*m*_ is the cell membrane capacitance, *I*_ion_ is the sum of the fourteen trans-membrane ionic currents and *I*_ext_ accounts for any externally applied current. This expression differs from that used in the model by Tong *et al*. [41] in that we include an additional gap-junction mediated coupling current, *I*_gap_. This term accounts for the current $I_{\text{gap}}^{p}$ induced by the interaction of myocytes with passive cells and, in the case of a 2-D lattice, the additional inter-myocyte coupling current $I_{\text{gap}}^{m}$. We use the standard convention where outward ionic currents and externally applied currents are taken as positive. For the purposes of the present study, we ignore the effect of external currents and set *I*_ext_ = 0.

The model by Tong *et al*. [41] also describes the evolution of the intra-cellular Calcium ion concentration, [Ca^2+^]_*i*_, in the cytosol,

$$\begin{array}{c} \frac{\text{d}{\left[{\text{Ca}}^{2+}\right]}_{i}}{\text{d}t}=-\left(J_{\text{Ca},\text{mem}}+J_{\text{PMCA}}+J_{\text{NaCa}}\right)\;, \end{array}$$ (3)

where the flux of Calcium ions has three components: (i) *J*_Ca,mem_, which represents Calcium flux from specific membrane channels, including L and T-types and other nonspecific cation currents; (ii) *J*_PMCA_, which represents the flux of plasmalemmal Ca^2+^-ATPase; and (iii) *J*_NaCa_, which represents flux from Na^+^-Ca^2+^ exchangers. The currents resulting from the plasmalemma and from the exchangers both extrude Calcium ions from the cell. In particular, the Na^+^-Ca^2+^ exchangers extract one Ca^2+^ ion from the cytosol for three Na^+^ ions pumped into the cell [45]. By their very nature, the ionic currents *J*_PMCA_ and *J*_NaCa_ must extrude Calcium, and as such, must be positive. Consequently, the current resulting from the action of the exchanger, *I*_NaCa_, is inward (or repolarizing) and hence, by the standard convention, negative. In our model we have ensured that the known physiological functions of the exchangers are cogently described, and that the requirements that *J*_NaCa_ ≥ 0 and *I*_NaCa_ ≤ 0 are satisfied. Additionally, motivated by the experimental literature (in particular [45]) as discussed in Sec. S.2 of the SI, we used different parameter values for the terms that describe the Na^+^-Ca^2+^ exchanger.

In order to motivate the electrical coupling mediated by gap junctions between myocytes and passive cells, we note the observation [20] that although ICLCs do not exhibit regular spontaneous depolarizations and appear unable to generate action potentials, the application of an external current causes their membrane potential to relax at a near-exponential rate with a characteristic time scale of ∼ 0.2 − 1*s* (see Fig. 7B of [20]). Thus, when a passive cell of this type is electrically coupled to a myocyte, its membrane potential dynamics can be described by:

$$\begin{array}{c} C_{P}\frac{\text{d}V_{P}}{\text{d}t}=G_{P}^{\text{int}}\left(V_{P}^{r}-V_{P}\right)+I_{\text{gap}}^{p}\;, \end{array}$$ (4)

where *C*_*P*_, $G_{P}^{\text{int}}$ and $V_{P}^{r}$ represent the capacitance, conductance and resting potential, respectively, of a generic passive cell and $I_{\text{gap}}^{p}$ is the coupling current. As a consequence, a myocyte with *n*_*p*_ passive cells in its neighborhood experiences a coupling current $I_{\text{gap}}=-n_{p}\;I_{\text{gap}}^{p}$. The current $I_{\text{gap}}^{p}$ is proportional to the difference between the potentials of the myocyte, *V*_*m*_, and passive cell, *V*_*P*_, across the electrically conducting pores that result from the existence of gap junctions. The coupling-induced current can hence be expressed as $I_{\text{gap}}^{p}=G_{p}\;(V_{m}-V_{P})$, where the gap junction conductance, *G*_*p*_, is directly related to the level of expression of the connexin proteins that constitute these junctions. As the conductance of a single gap junction channel has been estimated to be of the order of 50 pS [46], the relation between the conductance and then number of expressed gap junctions *n*_*gj*_ is simply *n*_*gj*_ ≈ *G*_*p*_/50 pS.

We note that although Eq. (4) is sufficient for our current purposes, it does not capture the full complexity of the passive cell membrane dynamics. As seen in Fig. 7B of [20], when the applied current is varied, the relaxation time scale changes, suggesting a dependence of $G_{P}^{\text{int}}$ as a function of the membrane potential. We further note that although Eq. (4) was formulated based on known properties of ICLCs, it can also be used to describe the behaviour of other electrically passive cells, such as fibroblasts, which have a membrane conductance of $G_{F}^{\text{int}}=1nS$ [47]. Indeed, it is instructive to consider the case where myocytes are simultaneously coupled to different types of passive cells (see Sec. S4 of the SI for more details).

**Fig 1:**
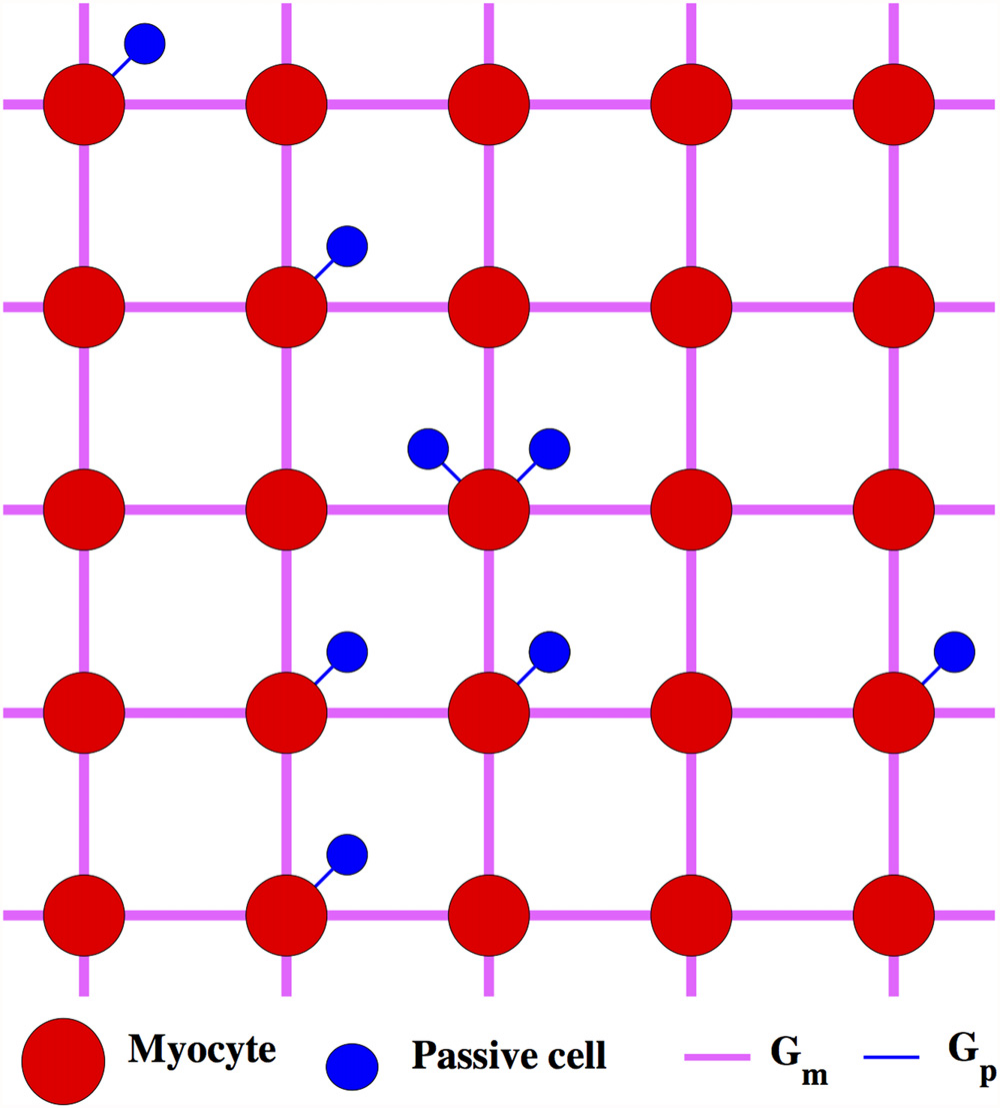
Schematic representation of the 2-D tissue. Schematic representation of the 2-D square lattice of uterine myocytes (shown in red), each myocyte coupled to a random number of passive cells (shown in blue). Neighboring myocytes are electrically coupled with strength *G*_*m*_ and the coupling strength between a myocyte and a passive cell is *G*_*p*_.

In order to describe the effect of inter-myocyte coupling, we assume that myocytes are coupled to their nearest neighbors on a 2-D square lattice of size *N* × *N*, and label each cell by the indices of its row (*a*) and column (*b*). In this case, each myocyte receives a coupling current $I_{\text{gap}}^{m}$ given by

$$\begin{array}{c} I_{\text{gap}}^{m}\left(a,b\right)=G_{m}\left(V_{m}\left(a+1,b\right)+V_{m}\left(a-1,b\right)+V_{m}\left(a,b+1\right)+V_{m}\left(a,b-1\right)-4V_{m}\left(a,b\right)\right)\;, \end{array}$$ (5)

where *G*_*m*_ is the conductance of the myocyte gap junctions. The total coupling current experienced by a myocyte coupled to both electrically passive cells, as well as other myocyte cells in a lattice (see Fig. 1) is thus $I_{\text{gap}}=-n_{p}\;I_{\text{gap}}^{p}+I_{\text{gap}}^{m}$. The coupling current Eq. (5) has the form of a diffusive term, with *G*_*m*_/*C*_*m*_ acting as an effective diffusion constant. We note that in the simple lattice, represented in Fig. 1, the distance between adjacent cells is expected to be of the size of the individual myocytes, which are known to be of the order of 100*μm*. In view of the very anisotropic shape of the cells [21], however, it is difficult to establish a very precise correspondence between the intercellular distance in the model and realistic properties of the tissue. This limitation does not affect the qualitative conclusions of the present work.

Finally, we note that one of the relaxation times *τ*_*α*_ can be of the order of several hundred pico seconds, and hence, the maximum simulation time step is greatly constrained. However, this very short time scale implies that the gating variable *x*_*α*_ relaxes very quickly to its steady-state value. Thus, in order to increase numerical efficiency, we make the assumption that *x*_*α*_(*t*) = *x*_*α*∞_(*V*_*m*_), which allows us to use a comparatively larger time step *dt* = 0.5*ms*. We have verified that the numerical results are not sensitive to this approximation, with only a 0.25% error in the periods of oscillation.

Apart from the details described above, our model equations and the parameter values used in our simulations correspond exactly to those used in Ref. [41]. Our numerical simulations involved the integration of the set of ordinary differential equations using a standard fourth-order Runge-Kutta scheme. For improved efficiency, the Message-Passing-Interface (MPI) library was implemented to distribute the load among up to 16 processors. When obtaining time series data, care was taken to ensure that any transient behaviour was discarded. While our model behaves slightly differently from the model by Tong *et al*. [41] in response to external stimuli (see Sec. S.3.2 of the SI), we emphasize that, as in Ref. [41], results obtained using our model are consistent with data from voltage clamp experiments (see Sec. S.3.1 of the SI for details of our validation tests). In addition, we have verified that our results are qualitatively robust, by simulating both the single cell and 2-D cases with slightly different sets of parameters, different realizations and, in the 2-D case, a larger lattice. We note that for a sufficiently large lattice size, the results do not qualitatively depend on *N*.

## Results

We now investigate the hypothesis mentioned in the introduction, namely that the interaction between coupled myocytes and passive cells is fundamental to their spontaneous activation during the late stages of pregnancy. As the number of gap junctions are known to increase during this period [24], one expects higher values for the effective coupling conductances *G*_*p*_ and *G*_*m*_. Moreover, although individual passive cell types are each characterized by a unique resting potential, a mixture of passive cell types can result in an effective $V_{p}^{r}$ that is different from those of the constituent cells. The effect of such phenomena on the dynamical behavior of the coupled system are shown below. In the following subsections, we present a numerical investigation of the electrical activity of myocyte cells coupled to *n*_*p*_ passive cells, followed by a study of the emergence of regimes of regular and irregular dynamical activity in a 2-D lattice of myocytes coupled to each other, as well as to passive cells.

### 3.1 Coupling a single myocyte to electrically passive cells

A myocyte is known to exhibit oscillatory behaviour when external current is injected into it (see SI for more details). As coupling through gap junctions with neighboring cells provides a source of such an inward current, the electrical state of a myocyte coupled to passive cells can be dynamically modulated by changing the strength of this coupling. When neighboring myocytes in a tissue are strongly coupled, as would be expected towards the late stages of pregnancy [24], their behaviour is sensitive to the average number of passive cells in the tissue—a property that we have explicitly verified through numerical simulations on an assembly of cells [48]. The limiting case of large coupling between myocytes can be approximated by considering a single myocyte coupled to *n*_*p*_ passive cells. Note that as *n*_*p*_ in this case effectively corresponds to the average number of passive cells in the tissue, it can take non-integer values. In the following, we investigate the dependence of the myocyte behaviour on the conductance of gap junctions between myocytes and passive cells, *G*_*p*_, the passive cell resting potential, $V_{p}^{r}$, and the average number of passive cells coupled to a myocyte, *n*_*p*_.

In our simulations, we have assumed that the myocyte membrane capacitance is *C*_*m*_ = 120*pF* and the passive cell has capacitance *C*_*P*_ = 80*pF* and conductance $G_{P}^{\text{int}}=1.0nS$. This is motivated by observations that the capacitance of rat myocytes is around 120*pF* during the late stages of pregnancy [21], and that the capacitance and input resistance of an ICLC are 84.8 ± 18.1*pF* and 3.04 ± 0.5*G*Ω, respectively [20]. Additionally, we assume that the sodium conductance is *g*_*Na*_ = 0.04*nS*/*pF*, which is in the range of values measured during late pregnancy (see [41] and references therein). We have verified that our results are robust with respect to small changes in the system parameters.

#### 3.1.1 Dependence on the gap junction conductance

**Fig 2:**
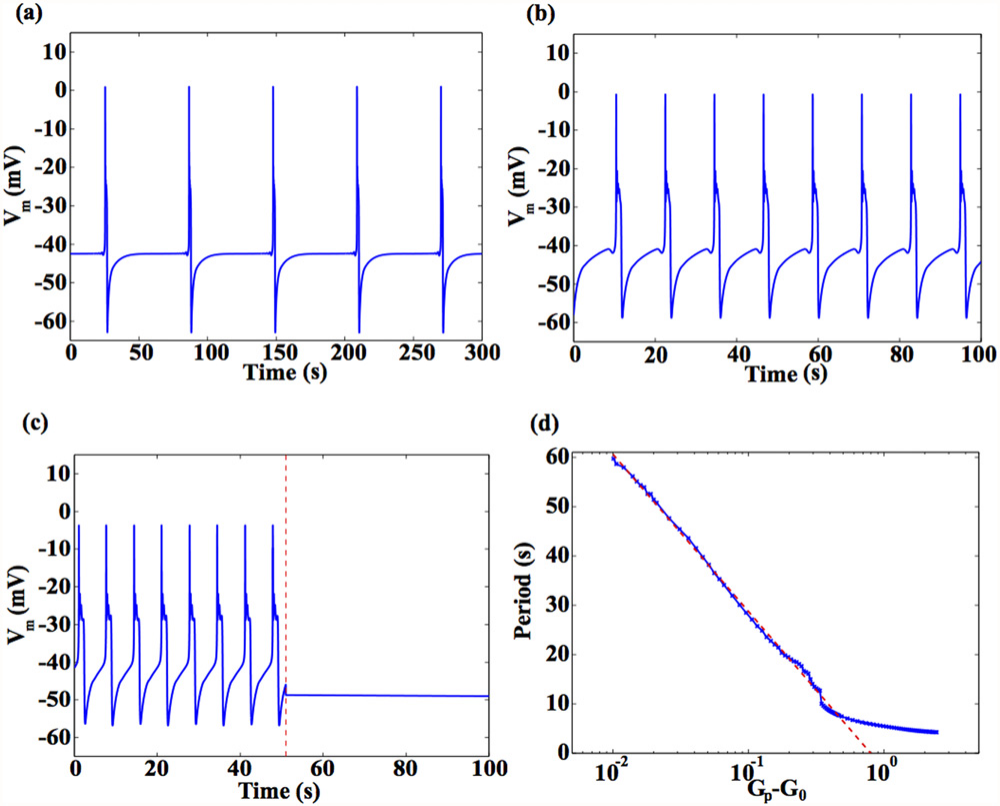
Possible dynamical regimes of a myocyte coupled to a passive cell. Dynamical behaviour of a myocyte coupled to a passive cell for different values of the gap junction conductance *G*_*p*_, and with $V_{p}^{r}=-35mV$ and *n*_*p*_ = 1. (a) At *G*_*p*_ = 0.174*nS*, the period is ∼ 1min. (b) At *G*_*p*_ = 0.5*nS*, the period has reduced to ∼ 12*s*. (c) At *G*_*p*_ = 1*nS*, the period reduces further to ∼ 7*s*. Here we observe that the sudden uncoupling of the myocyte and passive cell, at the time indicated by the vertical broken line, immediately terminates activity. (d) Above a critical value *G*_0_ = 0.164*nS*, we observe a logarithmic divergence in the oscillatory time period: *T* ∼ log[(*G*_*p*_ − *G*_0_)/*G*_0_] as indicated by the broken line.

The evolution of the membrane potential of a myocyte, *V*_*m*_, coupled to a single passive cell is displayed in Fig. 2 for different values of the gap junction conductance, *G*_*p*_. For values of *G*_*p*_ less than a critical threshold *G*_0_ ≈ 0.164*nS*, *V*_*m*_ approaches a steady state, while for values of *G*_*p*_ > *G*_0_, the electrical coupling induces spontaneous temporal oscillations whose time period decreases as *G*_*p*_ increases. As seen in Fig. 2a, the oscillation time period for *G*_*p*_ = 0.1741*nS* is *T* ∼ 1 min, while for *G*_*p*_ = 0.5*nS*, we find *T* ∼ 12*s* (Fig. 2b), and for *G*_*p*_ = 1*nS*, we find *T* ∼ 7*s* (Fig. 2c). As shown in Fig. 2c, the oscillatory behaviour can be suppressed immediately upon uncoupling the myocyte and the passive cell, i.e., by setting *G*_*p*_ to 0. This is consistent with the experimental observation that the addition of a gap junction uncoupler leads to rapid termination of electrical activity [27]. As *G*_*p*_ approaches *G*_0_ from above, the time period *T* grows like *T* ∼ log[(*G*_*p*_ − *G*_0_)/*G*_0_] (see Fig. 2d). Although this logarithmic divergence of time periods close to a critical point is suggestive of a homoclinic bifurcation [49], we note that the dynamical behaviour in the interface between the regimes of activity and inactivity is in fact more complicated. We observe that there exists a small range of values of *G*_*p*_ for which both stable and oscillatory solutions are possible. Depending on the precise choice of initial condition, the system may evolve to either of the two asymptotic solutions (attractors), corresponding to quiescence or oscillations.

#### 3.1.2 Dependence on the passive cell resting potential

**Fig 3:**
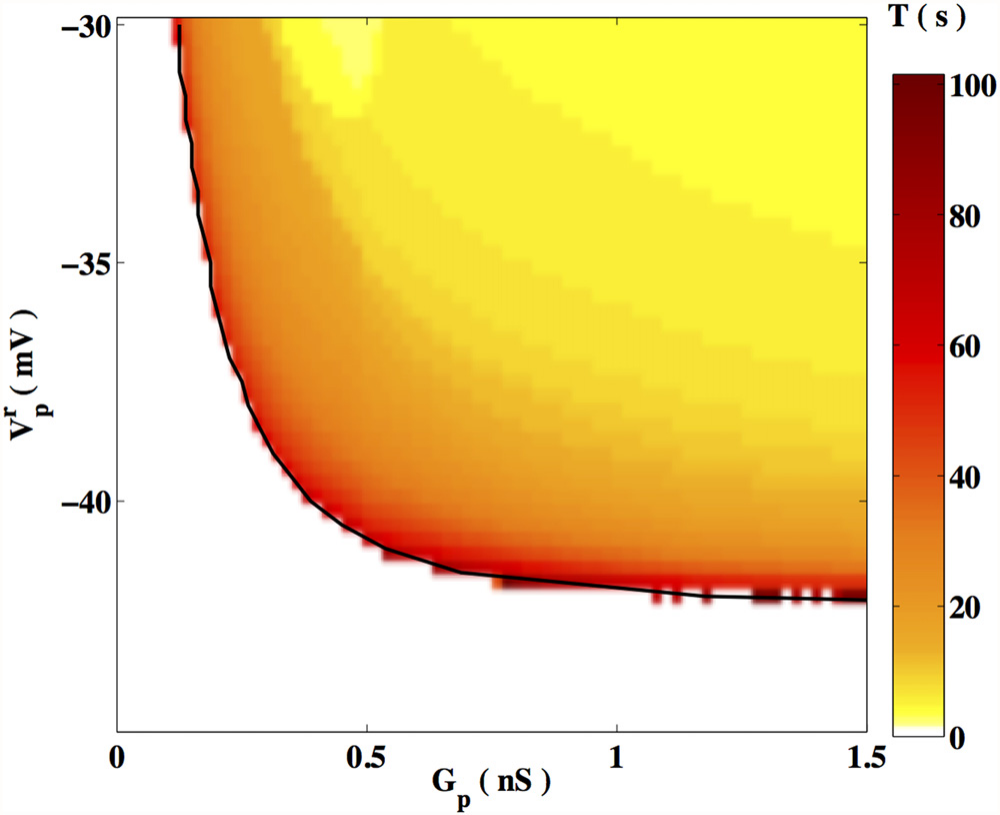
Time periods *T* of the oscillation as a function of coupling. The time period *T* (in sec) of the membrane potential, for a range of values of $V_{p}^{r}$ and *G*_*p*_. Regions shown in white correspond to the absence of oscillatory activity. The bifurcation from oscillatory activity to a quiescent dynamical regime occurs at the interface indicated by the continuous curve obtained by fitting numerical data.

The time periods, *T*, of the oscillations of the membrane potential, obtained for different values of the passive cell resting potential, $V_{p}^{r}$, and the gap junction conductance, *G*_*p*_, are displayed in Fig. 3. Consistent with the observations of Fig. 2d, the period of oscillation is very large close to a threshold value of *G*_*p*_ for values of $V_{p}^{r}$ larger than ∼ −42*mV*. In addition, *T* increases on decreasing $V_{p}^{r}$. For any given *G*_*p*_, there exists a threshold value of $V_{p}^{r}$ below which the solution approaches a steady, non-oscillating state. Conversely, as $V_{p}^{r}$ increases, one finds that *T* decreases, and can be as low as a few seconds for large values of $V_{p}^{r}$ and *G*_*p*_. As in the situation discussed in the previous subsection, the system can evolve to either a quiescent or oscillatory solution when $V_{p}^{r}$ and *G*_*p*_ are close to the bifurcation line (indicated in Fig. 3 by a continuous curve).

Fig. 3 suggests, in particular, that the gap junction conductance threshold is a decreasing function of $V_{p}^{r}$. This can be qualitatively understood by noticing that coupling a myocyte to a passive cell is equivalent to the addition of a current $I_{ext}∼-\frac{G_{P}^{\text{int}}G_{p}}{G_{P}^{\text{int}}+G_{p}}V_{p}^{r}$ in the expression for myocyte membrane potential, Eq. (2), at least in the limit of large passive cell relaxation time. From this expression for the external current *I*_*ext*_, elementary algebraic considerations show that as the potential $V_{p}^{r}$ increases, the coupling *G*_*p*_ necessary to deliver a given current decreases (see also Supplementary Information).

#### 3.1.3 Dependence on the number of passive cells

**Fig 4:**
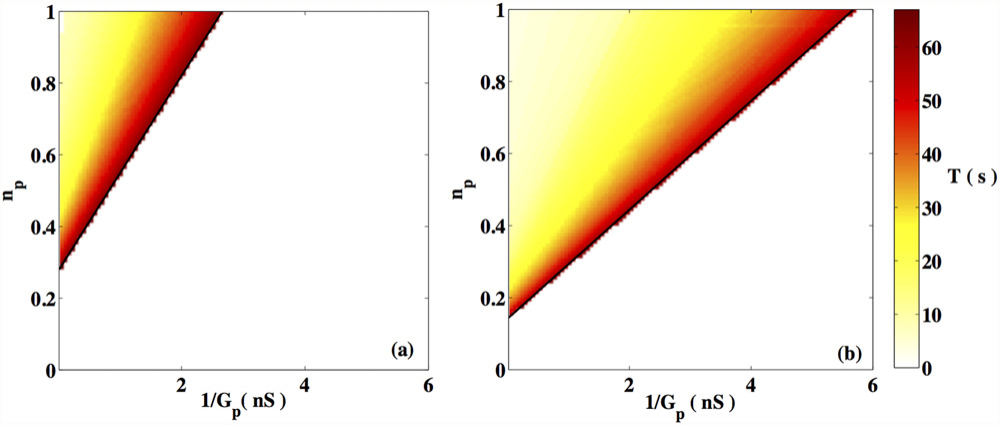
Time periods *T* as a function of the number of coupled passive cell. Time periods of oscillation, *T* (in sec) at two different values of $V_{p}^{r}$, for a range of values of *n*_*p*_ and *G*_*p*_. Regions shown in white correspond to the absence of oscillatory activity. Activity is seen when *n*_*p*_ ≥ *A* + *B*/*G*_*p*_ (indicated by the solid line), a functional form that can be justified from elementary considerations, see text. (a) $V_{p}^{r}=-40mV$ (*A* ≈ 0.28, *B* ≈ 0.27) (b) $V_{p}^{r}=-35mV$ (*A* ≈ 0.17, *B* ≈ 0.15).

It is known that the total number of passive cells in uterine tissue is only a fraction of that of the myocytes [19]. As mentioned earlier, each myocyte is attached to *n*_*p*_ passive cells. Fig. 4 displays the domain of oscillatory activity in the (*G*_*p*_, *n*_*p*_) plane, observed for two different choices of the passive cell resting potential: $V_{p}^{r}=-40mV$ (Fig. 4a) and $V_{p}^{r}=-35mV$ (Fig. 4b). In each case, we find that the cell is quiescent for low values of *G*_*p*_ and *n*_*p*_, while spontaneous oscillatory activity is generated on increasing these parameters. We note from Fig. 4 that for any given value of *G*_*p*_ there exists a critical value of *n*_*p*_ below which spontaneous oscillations will not occur. In the limit 1/*G*_*p*_ → 0, we find this critical value to be *n*_*p*_ ≈ 0.28 (*n*_*p*_ ≈ 0.17) for a passive cell resting potential $V_{p}^{r}=-40mV$ ($V_{p}^{r}=-35mV$).

We observe that the curve delimiting the region of activity in Fig. 4 has a simple analytic form *n*_*p*_ = *A*/*G*_*p*_ + *B*, where the parameters *A* and *B* are related by $B=A/G_{P}^{\text{int}}$. In the limit where the passive cell resistivity $G_{P}^{\text{int}}$ is large, the passive cell membrane potential relaxes quickly to its equilibrium value. From Eqs. (2) and (4) one can show that the coupling term in Eq. (2) is equivalent to adding an external current $I_{ext}∼-n_{p}\frac{G_{P}^{\text{int}}G_{p}}{G_{P}^{\text{int}}+G_{p}}V_{p}^{r}=-n_{p}/\left(\frac{1}{G_{P}^{\text{int}}}+\frac{1}{G_{p}}\right)V_{p}^{r}$. This expression implies that the effect of the coupling in the equation for the myocyte action potential depends on the quantity $n_{p}/\left(\frac{1}{G^{int_{P}}}+\frac{1}{G_{p}}\right)$, which is constant provided *n*_*p*_ is proportional to $1/G_{P}^{\text{int}}+1/G_{p}$. Hence the analytical form for *n*_*p*_ stated above delineates the region of parameter space where oscillations occur (Fig. 4).

### 3.2 Coupling myocytes to passive cells in a 2-D lattice

We now investigate dynamical patterns of activity that arise on a spatially extended domain characterized by nearest-neighbor interactions between the myocytes. To this end, we assume that myocytes are electrically coupled in an *N* × *N* square lattice (Fig. 1); we take *N* = 50 in the present study. Additionally, each myocyte is coupled to an integer number of passive cells *n*_*p*_ drawn from a random distribution *P*(*n*_*p*_), chosen here to be binomial with mean *f* = < *n*_*p*_ > = 0.2. Numerical simulations reveal that the precise choice of boundary conditions for our model can affect some qualitative features of the observed patterns. In this paper, we limit our 2-D investigation to the study of the dynamics in an isolated segment of late-pregnant myometrium by imposing no-flux boundary conditions on *V*_*m*_, thus allowing for direct comparisons with experiments, such as those performed by Lammers and coworkers [44, 50, 51]. In the following, we set *G*_*p*_ = 3.5*nS* and systematically vary the inter-myocyte gap junction conductance *G*_*m*_.

#### 3.2.1 Dependence on the inter-myocyte gap junction conductance

**Fig 5:**
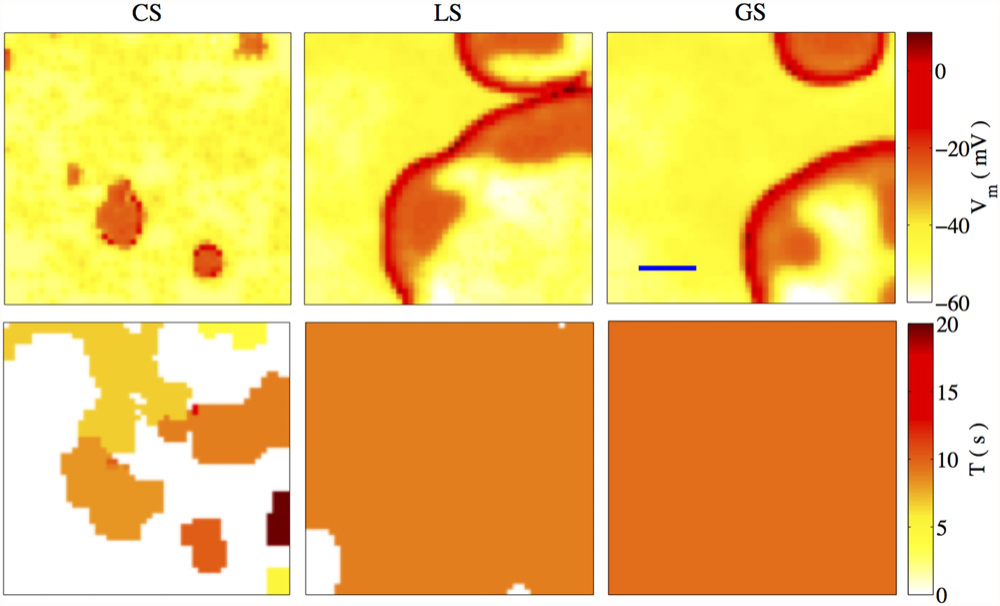
Patterns of electrical activity. Pattern observed for different coupling strengths *G*_*m*_ between myocytes on a 50 × 50 lattice, where each myocyte interacts on average with *f*(= 0.2) passive cells. The upper row shows snapshots of the membrane potential while the lower row shows the corresponding effective time periods of oscillatory activity. The horizontal bar in the upper right-most panel indicates the length corresponding to 10 cells, which is of the order of ∼ 1 − 2*mm*. (a) Cluster Synchronization (CS) is observed at *G*_*m*_ = 0.48*nS* where cells group into several synchronously oscillating clusters, each characterized by a different frequency, coexist with regions in which the tissue is at rest. (b) At *G*_*m*_ = 1.8*nS* all cells in the lattice that oscillate do so with a single frequency. However, we also observe a few non-oscillating cells indicating that this corresponds to the LS regime. (c) At *G*_*m*_ = 2.4*nS*, which lies in the GS regime, every cell in the lattice oscillates with the same frequency.

The dependence of the electrical activity of the lattice on the inter-myocyte gap junction conductance *G*_*m*_ is shown in Fig. 5. As the coupling strength is known to increase during pregnancy [24], these results are plausibly indicative of the transition towards coherent activity in the uterus close to term.

For low values of *G*_*m*_, a significant number of cells remain quiescent, while the remaining cells organize themselves into a few localized clusters, each of which are characterized by a unique oscillating frequency. This regime, referred to as cluster synchronization (CS), is shown in Fig. 5 (left column), where different clusters with characteristic effective oscillatory periods in the range 5 – 10 sec are observed for *G*_*m*_ = 0.48*nS*. It has been shown, using a simplified model of myocyte activity [48], that the electrical activity originates in regions where the coarse-grained density of passive cells attached to the myocytes is high.

On increasing *G*_*m*_, we observe that the various clusters begin to merge, eventually giving rise to a scenario where all the cells that oscillate do so with the same frequency (see the middle column of Fig. 5, which displays results for *G*_*m*_ = 1.8*nS*). In this regime, referred to as local synchronization (LS), it is appropriate to define the fraction of oscillating cells, by *n*_*osc*_ = *N*_*osc*_/*N* ^2^ where *N*_osc_ is the number of oscillating cells [36]. We find the existence of pockets of cells that remain quiescent, and also observe that regions with a high coarse-grained density of passive cells produce travelling waves that propagate through the system, thus effectively acting as “pacemaker regions” [48].

As we increase *G*_*m*_ further, we find that every cell in the system oscillates at exactly the same frequency (see the right column of Fig. 5, which displays results for *G*_*m*_ = 2.4*nS*). This dynamical state is known as global synchronization (GS). The transition from LS to GS is characterized by an increase in the fraction of oscillating cells *n*_*osc*_ to 1.

**Fig 6:**
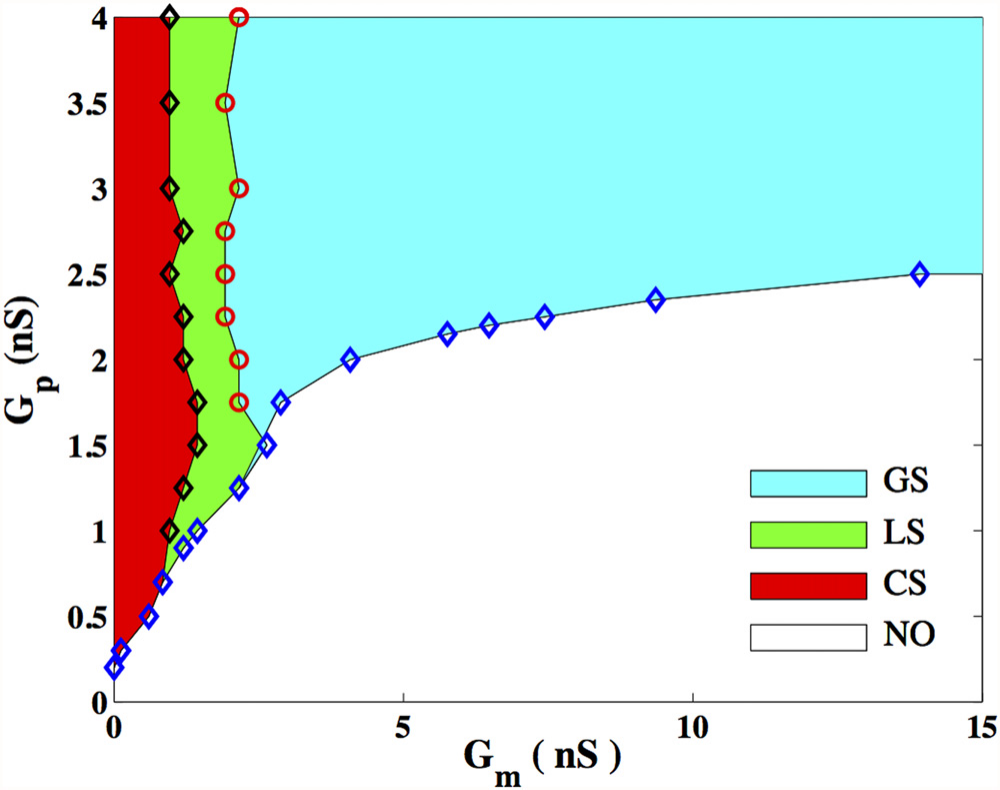
Phase space describing the dynamical systems of the model. Dynamical regimes observed in the 2-D lattice of coupled myocytes and passive cells for a range of coupling strengths. Three distinct synchronization regimes are observed: CS at low *G*_*m*_, LS at intermediate *G*_*m*_ and GS at high *G*_*m*_. For every value of *G*_*m*_, there exists a critical value of *G*_*p*_ below which no oscillations (NO) are observed. The symbols indicate numerically determined points lying on the boundaries between the various dynamical regimes.

The phase diagram in Fig. 6, obtained over a range of values of *G*_*m*_ and *G*_*p*_, displays the aforementioned regimes of dynamical behaviour, viz., CS, LS and GS. An additional state is seen for sufficiently low *G*_*p*_ and sufficiently large *G*_*m*_ where no oscillations (NO) are observed. We observe in Fig. 6 that when *G*_*m*_ is large, the transition between NO and GS occurs at a value of *G*_*p*_ ≈ 2.7*nS*, consistent with Fig. 4. The phase diagram in Fig. 6 is qualitatively similar to that obtained with the simpler FitzHugh-Nagumo model, used for describing the dynamics of an excitable cell [36]. We note that for a range of parameter values in the domain corresponding to GS in Fig. 6, the activity is in fact irregular, an issue discussed in Sec. 3.2.2.

**Fig 7:**
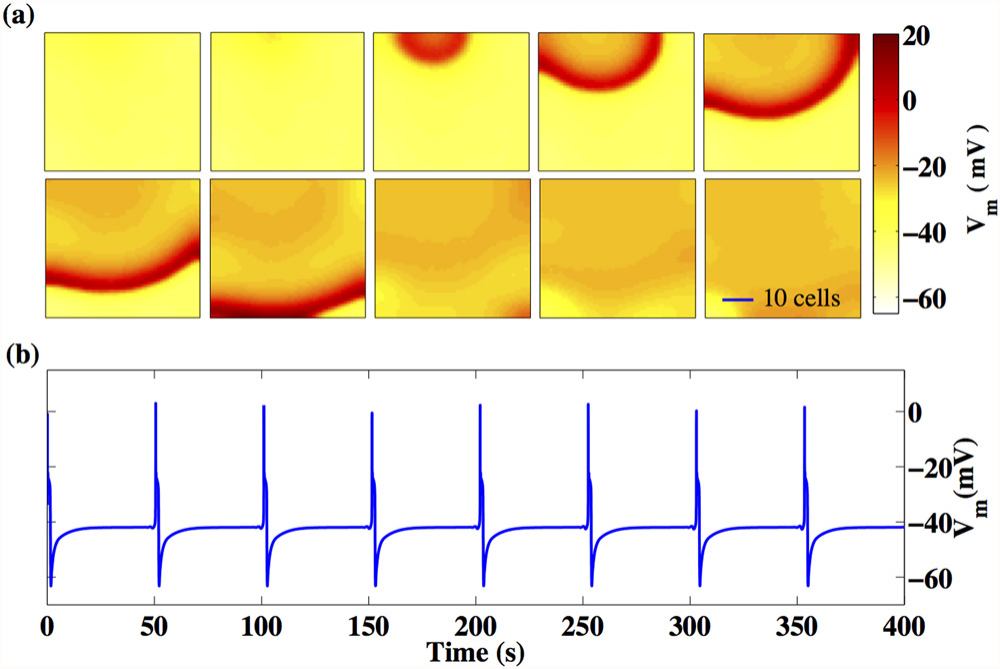
Regular periodic activity in the 2-D lattice of coupled myocytes and passive cells. The regime presented here corresponds to an inter-myocyte coupling strength, *G*_*m*_ = 20*nS*. (a) Waves are emitted periodically from a single, dominant region characterized by high passive cell density. The snapshots are separated in time by 75 ms. The horizontal bar in the lowest, right-most panel indicates the length corresponding to 10 cells, which is of the order of ∼ 1 − 2*mm*. (b) This behaviour causes each cell in the system to exhibit a periodic pattern of activity with a period *T* ∼ 50 s. The only difference between the recorded time series of any two cells in the system is a temporal shift, dependent on the proximity of the cell to the source.

For a sufficiently large inter-myocyte gap junction conductance (*G*_*m*_ ≳ 19.2*nS*) the activity of the system is characterized by a simple, regular spatial pattern (Fig. 7). Here, an action potential is emitted in the form of a target wave from a single, dominant region of high passive cell density, which effectively acts as a local pacemaker. The nature of the transition from distinct, competing wave sources at low *G*_*m*_ to a single dominant source at large *G*_*m*_ is explicated in Ref. [48]. The range of values of *G*_*m*_ spanned in Fig. 6 correspond to experimentally relevant values of inter-cellular coupling in the uterine myometrium [24]. Based on the results of Ref. [36], we expect that coherent activity over the entire system will be observed at even larger values of *G*_*m*_.

#### 3.2.2 Emergence of irregular activity

Despite the fact that the activity of all oscillating cells in the LS and GS regimes have the same effective time period, we note that this does not imply that the activity of all cells are temporally synchronized in these regimes, nor that they exhibit simple dynamical behaviour. In fact, for values of the inter-myocyte gap junction conductance in the range 6*nS* ≲ *G*_*m*_ ≲ 19.2*nS* the region of parameter space characterized by GS occasionally exhibits irregular activity: oscillations with a period of ∼ 15*s* that are erratically interrupted, for short durations, by oscillations with a period of ∼ 1*s*. This activity arises due to the competition between strong and weak pacemaker-like regions that can, on occasion, generate disordered activity in the form of transient spiral waves. The strength and number of pacemaker-like regions is strongly correlated to the passive cell distribution on the lattice, and the possibility of competition between regions is more significant for larger lattices [48].

**Fig 8:**
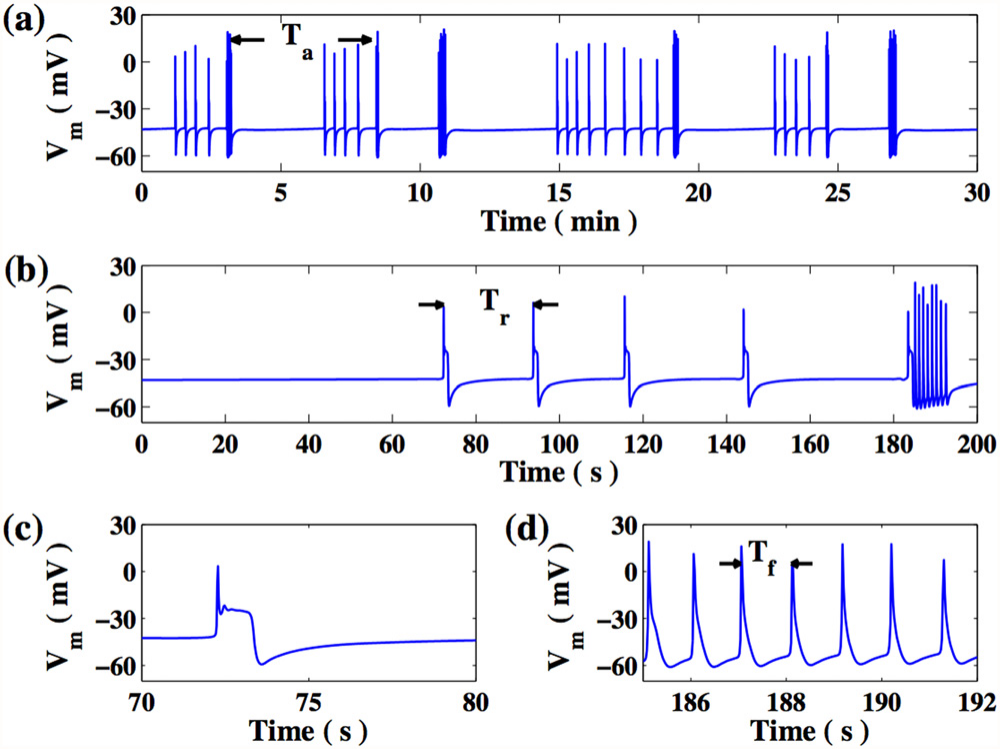
Irregular patterns of activity in the 2-D lattice of coupled myocytes and passive cells. The regime observed here corresponds to an inter-myocyte coupling strength, *G*_*m*_ = 12*nS*. (a) The membrane potential of a single cell exhibits recurrent patterns of activity, each pattern arising after an interval *T*_*a*_. (b) Each pattern is characterized by an initial quiescent phase, followed by a series of action potentials with period *T*_*r*_ ∼ 20*s* and a brief duration of fast oscillations with periods *T*_*f*_ ∼ 1*s*. The profiles of a representative action potential (c) and fast oscillations (d) are also shown.

As this irregular behaviour occurs in the GS regime, all cells exhibit the same qualitative time series, with only a temporal shift. Hence, we restrict our attention to the behaviour of a generic cell in the lattice. The evolution of the membrane potential of a single randomly selected cell for *G*_*m*_ = 12*nS* is shown in Fig. 8. We observe periodically occuring patterns of irregular activity, with consecutive patterns separated by *T*_*a*_ ∼ 4 min. The structure of this irregular pattern is shown in Fig. 8 (b), where a sequence of action potentials with a period *T*_*r*_ ∼ 20*s* are followed by a set of fast oscillations of period *T*_*f*_ ∼ 1*s*. As seen from Fig. 8 (c-d), the fast oscillations do not exhibit the “plateau” that characterizes action potentials. We find that the initial regular activity (of period *T*_*r*_) arises from waves generated by a single, dominant “pacemaker” region which, as suggested by the detailed analysis of a simplified model [48], is characterized by a high density of passive cells.

**Fig 9:**
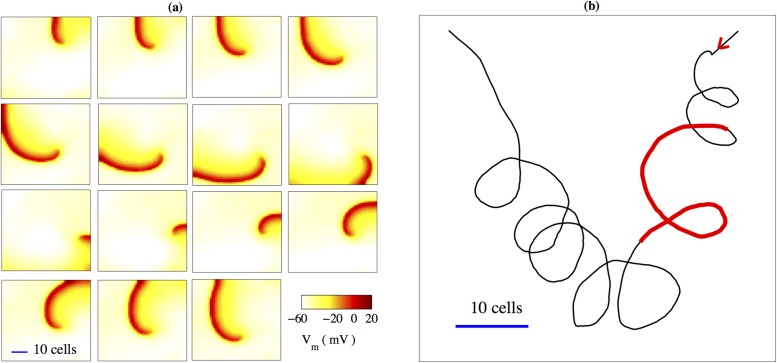
Spiral wave activity. Occurrence of spiral waves of activity in a 50 × 50 lattice of coupled myocytes and passive cells, observed for inter-myocyte coupling strength, *G*_*m*_ = 12*nS*. (a) Snapshots of membrane potential are shown at intervals of *T* = 100*ms*, in sequence from left to right and from top to bottom. (b) Trajectory indicating the motion of the tip of the spiral on the 2-D lattice. The two arrows indicate the locations of the spiral when it emerges and disappears, respectively The thick segment corresponds to the sequence shown in (a). The horizontal bars in the lowest, left-most panel of (a), and in (b) indicate the length corresponding to 10 cells, which is of the order of ∼ 1 − 2*mm*.

The irregular behaviour is a consequence of transient, recurrent spiral wave activity (Fig. 9). The motion of the spiral wave can be characterized by the trajectory of its tip, which is defined here as the position on the lattice where the membrane potential is *V*_*m*_ = −30*mV*, intermediate between resting and depolarized states, and where the variable *h*, describing the inactivation of sodium channels is equal to *h* = 0.5. The motion of the spiral tip over a single rotation period is shown in Fig. 9 b. We observe that the emergence of spiral activity is not sensitive to the precise choice of model parameters.

**Fig 10:**
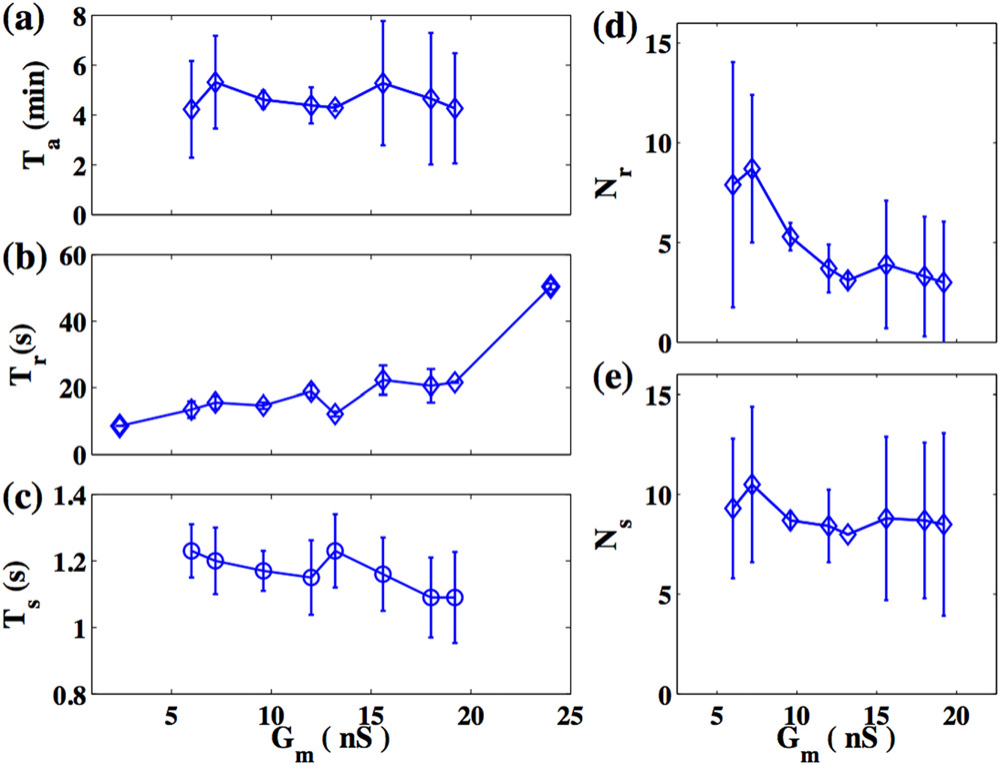
Characterisation of the irregular regimes. Dependence on *G*_*m*_ of the quantities characterizing the patterns of activity shown in Fig. 8, namely the mean values of (a) *T*_*a*_, (b) *T*_*r*_, and (c), *T*_*f*_ (c); and of the number of (d) action potentials, *N*_*r*_, and (e) fast oscillations, *N*_*f*_. The error bars indicate the standard deviation of the fluctuations of each individual quantity. Each quantity is measured over an interval of at least 6000 s. The quantities shown here have been determiend after averaging over 30 independent realizations of the passive cell distribution.

To quantitatively characterize the spiral dynamics, we identify *T*_*f*_ as the rotation period of the spiral and *T*_*a*_ as the interval between two successive appearances of transient spiral activity. Additionally, *T*_*r*_ is identified as the interval between successive regular waves generated by the region with higher passive cell density. We also define *N*_*r*_ as the number of regular waves appearing prior to the appearance of a spiral and *N*_*f*_ as the number of rotations by a spiral during its lifetime. Fig. 8 shows that the values of the characteristic times *T*_*a*_, *T*_*r*_ and *T*_*f*_, as well as *N*_*r*_ and *N*_*f*_, vary between successive irregular patterns. Nevertheless, simulations over large time scales confirm that the behaviour shown in Fig. 8 is statistically stationary. Fig. 10 displays the dependence of the mean of these quantities, obtained over a sufficiently long time interval, on *G*_*m*_, with the error bars indicating the standard deviation. We observe that the mean value of *T*_*a*_ is approximately constant over the range 6*nS* ≤ *G*_*m*_ ≤ 19.2*nS*. In contrast, the mean value of *T*_*r*_ (*T*_*f*_) slightly increases (decreases). Despite the relatively large error bars for *N*_*r*_ and *N*_*s*_, we find that these quantities remain more or less constant, except for a decrease in *N*_*r*_ at low *G*_*m*_.

## Discussion

In this paper we address the question as to how spontaneous contraction-inducing currents can emerge in the uterus despite the absence of any clearly identifiable pacemaker cells in this organ. Our proposed solution is motivated in part by the experimental observation that gap junction expression strongly increases towards the end of pregnancy [23, 24], leading to an increase in inter-cellular coupling. We investigate the role of such coupling in triggering spontaneous uterine activity by considering the coupling of excitable myocyte cells to electrically passive cells in a mathematical model. We have used a realistic description of myocyte activity based on a model recently developed by Tong *et al*. [41], and have incorporated interaction between myocytes and passive cells. We investigate the collective activity of such an assembly both in the context of a single myocyte coupled to one or more passive cells, as well as a 2-D lattice of myocytes with nearest neighbor coupling with each myocyte interacting with a randomly distributed number of passive cells. While simplified models of excitable cells interacting with passive cells have been considered in earlier investigations [36], the use of physiologically realistic model in the present paper allows us to make semi-quantitative predictions and provides a framework for explaining uterine tissue activity both *in vitro* and *in vivo*.

In addition to the gap junctional expression, several other physiological parameters, such as the sodium conductance, *g*_Na_, have been observed to change significantly through the course of pregnancy [21]. The experimentally observed values of the resting potential of Interstitial Cajal-like cells (ICLC), as well as of myocytes, are also found to vary over a wide range. For the purposes of our simulations, we choose a set of parameters over a range well within the bounds set by experimental observations. We have confirmed that the results of our model simulations are qualitatively robust to small variations in parameter values (see SI for details).

We find that the properties of a single myocyte coupled to a number of passive cells are qualitatively very similar to the behaviour observed using a simple excitable model description of myocyte activity [36]. The coupling between myocyte and passive cells, *G*_*p*_ has been chosen to be of the order of ∼ 1*nS*, which corresponds to the expression of ∼ 20 gap junctions of conductance ∼ 50 pS [46]. We note that this is realistic, given the number of gap junctions known to be expressed in a myocyte [24].

We observe that the quantitative nature of our numerical results is dependent on the precise value of the passive cell resting potential, $V_{p}^{r}$, as can be seen from Fig. 3. It has been experimentally observed that the resting potential of an ICLC is −58 ± 7 mV. As the fraction of ICLCs in the tissue does not exceed *n*_*p*_ ≈ 0.2 [14, 19], the results shown in Fig. 4 suggest that no oscillations would occur in a tissue containing only myocytes and ICLCs. However, uterine tissue contains other electrically passive cells, such as fibroblasts which have a much higher resting potential, $V_{p}^{F}\approx -15mV$, and are known to play an important structural role in uterine tissue [16, 17]. A theoretical analysis, documented in the Supplementary Information, suggests that a small population of such passive cells may result in an “effective” resting potential for the passive cells that is higher than that of ICLCs alone. This provides a justification for the range of values of $V_{p}^{r}$ used in the simulations reported here, which exhibit spontaneous oscillations in a system of coupled myocytes and passive cells.

For the situation in which myocytes are coupled on a 2-D lattice to their nearest neighbors, as well as to a random number of passive cells, we observe that a progressive increase in the coupling between cells results in a gradual transition from quiescence to the appearance of small clusters of oscillating cells. Further increase in coupling causes these clusters to grow and merge until a single cluster occupies the entire system. These features are qualitatively consistent with observations from experiments on a co-culture of myocytes and fibroblasts [35]. This transition to regular global synchronization occurs via an interesting dynamical regime in which transient, recurrent spiral waves propagate through the system giving rise to activity with a period of ∼ 1*s*. This regime of irregular spatiotemporal behaviour has not been previously reported. It is of interest to note that these patterns resemble the complex waves seen in *in vitro* experiments on guinea pig uterine tissue performed by Lammers *et al* [44]. Thus, although a precise quantitative comparison between simulations and experiments would require a more exhaustive investigation, our results are in close qualitative agreement with the observed features of waves propagating in uterine tissue.

We find that it takes approximately 340*ms* for a wave to propagate across a 2-D myocyte assembly of size 50 × 50 cells. Assuming that the length of a cell is ∼ 225*μm* [21], this corresponds to an action potential propagation velocity of ≈ 3.3*cm*/*s*, which is consistent with the values obtained in Ref. [44, 51]. We observe that the period of fast activity is of the order of ∼ 1*s*, which is also in agreement with the results of Ref. [44]. Additionally, we note that the value of the inter-myocyte coupling used to obtain the patterns shown in Figs. 8 and 9 is *G*_*m*_ = 12*nS*, which corresponds to an expression of *n*_*gj*_ ≈ 240 gap junctions, a value consistent with experimental observations [24].

For medical applications, the key question is to understand the generation of force in the uterine tissue. In myocytes, action potentials induce a large influx of Calcium, which in turn leads to cell contraction. Available models addressing the question of force generation rest on bursts of Calcium influx inside cells [41, 43, 52]. It is an open question as to whether such activity is a result of intrinsic electrophysiological dynamics of local cell clusters or due to re-entrant waves propagating around the organ. We note that in our simulation of a 2-D lattice of coupled myocytes and passive cells, rapid spiking activity is observed when the system exhibits spiral waves (Figs. 8 and 9). Determining whether spirals induce transient, pathological contractions, as is the case in the heart, or are required to generate a strong force at the time of delivery, cannot be answered without a better understanding, both at the cellular and tissue level [9, 44].

The results reported in this paper present a picture that is qualitatively, and to an extent quantitatively, consistent with a number of experimental observations, despite the limitations inherent to physiologically detailed models, such as the uncertainties in the characterization of potentially crucial model parameters. This suggests that the mechanism under consideration, namely the electrical coupling between excitable myocytes and passive cells, is at least partially responsible for the generation of spontaneous electrical activity. Our work provides a feasible and falsifiable hypothesis that suggests new avenues for further investigation into this issue, such as the effect of increasing sodium conductance, or the role of hormones such as oxytoxin in the course of pregnancy.

## Supporting Information

S1 Table Parameters used in the model. Values of the parameter used in the description of the Na^+^-Ca^2+^ exchanger. The description of the Na^+^-Ca^2+^ exchanger in Tong *et al*. [41] was based on the approach of Weber *et al*. [45], and we use the latter values in the current work, with the exception of K_*m*,*Allo*_, whose value was chosen to lie between the corresponding values used in Weber *et al*. [45] and that displayed in the SI of Tong *et al*. [41].
(PDF)

S1 Fig Simulated voltage-clamp experiments on Ca^2+^ channels. (a)-(b) Behaviour of L-type Ca^2+^ channel current, *I*_*CaL*_, for different depolarizing potentials in the range −40 mV to 0 mV at voltage steps of 10 mV with a holding potential *V*_*h*_ = −60mV, shown both as a function of (a) time and (b) depolarizing potential *V*_*d*_, superimposed with results obtained using the model of Tong *et al*. [41]. (c) Behaviour of T-type Ca^2+^ channel current, *I*_*CaT*_, for different depolarizing potentials in the range −60 mV to 20 mV, with a holding potential *V*_*h*_ = −80mV.
(TIFF)

S2 Fig Simulated voltage-clamp experiments on Na^+^ and K^+^ channels. (a) Behaviour of Na^+^ channel current, *I*_*Na*_, at different depolarizing potentials in the range −40 mV to 20 mV with a holding potential *V*_*h*_ = −40 mV. (b) Behaviour of the K^+^ channel current *I*_*K*1_ for *g*_*k*_ = 0.8*nS*/*pF*, at different depolarizing potentials in the range −40 mV to 10 mV with a holding potential *V*_*h*_ = −80 mV, normalized to the peak current at 10 mV. (c) Behaviour of the total K^+^ channel current, at different depolarizing potentials in the range −30 mV to 70 mV with a holding potential *V*_*h*_ = −80 mV, normalized to the peak current at *V*_*d*_ = 70 mV.
(TIFF)

S3 Fig Simulated action potentials. Action potentials using the model used in this article, compared with the corresponding results obtained using the model of Tong *et al*. [41], for the situations where: (a) A depolarizing current clamp of amplitude *I*_*st*_ = −0.5*pA*/*pF* is applied for two seconds under control conditions (c.f. Figure 12 of Tong *et al*. [41]). (b) A stimulus of amplitude −1.5 pA/pF is applied over 20 ms at 0.4 Hz (c.f. Figure 13 of Tong *et al*. [41]).
(TIFF)

S4 Fig Response to a constant stimulus. Behaviour of the model of Tong *et al*. [41], for the situation where a constant stimulus (*I*_*st*_ = −0.1 pA/pF) is applied for two values of the sodium conductance, viz. (a) *g*_*Na*_ = 0 nS/pF, and (b) *g*_*Na*_ = 0.04 nS/pF. The evolution of the [top] membrane potential, and [bottom] intracellular calcium concentration is displayed in each case. The vertical dashed line indicates the time at which the stimulus is turned off.
(TIFF)

S5 Fig Response to a constant stimulus after the stimulating current is turned off. Behaviour of our model for the situation where a constant stimulus (*I*_*st*_ = -0.1pA/pF) is applied. When the current is turned off (at the time indicated by vertical dashed line), the oscillations cease, and the system eventually returns to its resting state.
(TIFF)

S6 Fig Decay rates of the coupled system myocyte-passive cell. Decay rates *λ*_1,2,3_ of membrane potentials *V*_*m*_, *V*_*I*_ and *V*_*F*_, respectively. When *C*_*m*_ (*C*_*I*_) is changed by 50%, while leaving *C*_*F*_ and *C*_*I*_ (*C*_*m*_) unchanged, it can be seen that the fibroblast has the largest decay rate that is one order of magnitude larger than the others. The method is applied to determine the decay rate of the other cells.
(TIFF)

S7 Fig Eigenvectors corresponding to the relaxation of the system myocyte-passive cell. Components of eigenvectors associated with (a) *λ*_1_, (b) *λ*_2_ and (c) *λ*_3_.
(TIFF)

S8 Fig Phase space for a coupled system myocyte-passive cell. The (*n*_*p*_, *G*_*p*_) parameter space for the case of a single myocyte coupled to *n*_*I*_ ICLCs and *n*_*F*_ fibroblasts, indicating the region where oscillations are observed. The ratio of fibroblasts to ICLCs, *nF*:*nI* is 1:9. For comparison with the results of coupling a myocyte with an effective passive cell see Fig. 4 in the main text.
(TIFF)
