# Supplementary material for: The Role of Cellular Coupling in the Spontaneous Generation of Electrical Activity in Uterine Tissue
Source: PLoS One. 2015 Mar 20;10(3):e0118443. doi: 10.1371/journal.pone.0118443 (PMC4368634; doi:10.1371/journal.pone.0118443)
Supplement: S1 Table — Values of the parameter used in the description of the Na+-Ca2+ exchanger. The description of the Na+-Ca2+ exchanger in Tong et al. [41] was based on the approach of Weber et al. [45], and we use the latter values in the current work, with the exception of Km,Allo, whose value was chosen to lie between the corresponding values used in Weber et al. [45] and that displayed in the SI of Tong et al. [41]. (PDF) [file pone.0118443.s001.pdf]

Variables	Tong *et al.* [1] (SI)	Tong *et al.* [1] (code)	Weber *et al.* [2]	Present work
$\bar{J}_{NaCa}$(pA/pF)	11.67	11.67	22.6	22.6
$K_{m,Allo}$(mM)	3×10^{-3}	3×10^{-4}	1.25×10^{-4}	1.25×10^{-3}
$K_{m,\text{Nai}}$(mM)	30	30	12.3	12.3
$K_{m,\text{Cai}}$(mM)	0.007	0.007	0.0036	0.0036
n_{Allo}	4	4	2	2

Table S1: Values of the parameter used in the description of the Na^+-Ca^{2+} exchanger. The description of the Na^+-Ca^{2+} exchanger in Tong *et al.* [1] was based on the approach of Weber *et al.* [2], and we use the latter values in the current work, with the exception of $K_{m,Allo}$, whose value was chosen to lie between the corresponding values used in Weber *et al.* [2] and that displayed in the SI of Tong *et al.* [1].

References

[1] Tong WC, Choi CY, Karche S, Holden AV, Zhang H, et al. (2011) A computational model of the ionic currents, Ca^{2+} dynamics and action potentials underlying contraction of isolated uterine smooth muscle. PLoS ONE 6: e18685.

[2] Weber CR, Ginsburg KS, Philipson KD, Shannon TR, Bers DM (2001) Allosteric regulation of Na/Ca exchange current by cytosolic Ca in intact cardiac myocytes. J Gen Physiol 117: 119–132.
