# Supplementary figures and images for: The Role of Cellular Coupling in the Spontaneous Generation of Electrical Activity in Uterine Tissue
Source: PLoS One. 2015 Mar 20;10(3):e0118443. doi: 10.1371/journal.pone.0118443 (PMC4368634; doi:10.1371/journal.pone.0118443)

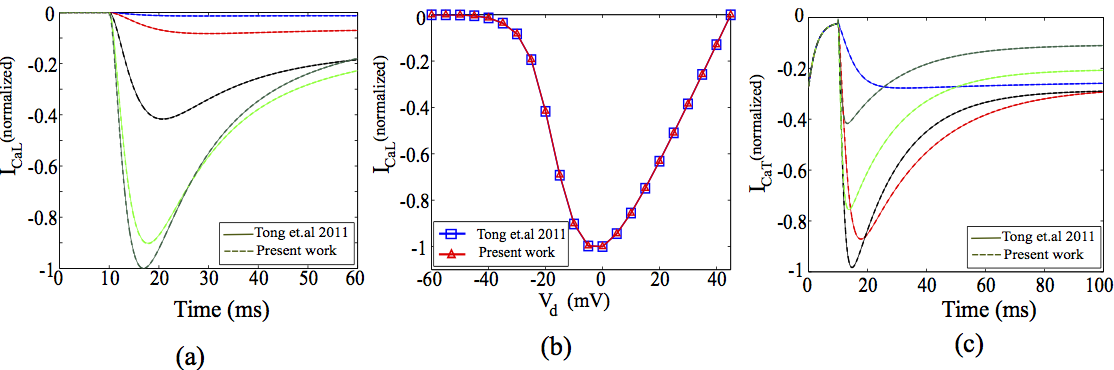

Supplement: S1 Fig — (a)-(b) Behaviour of L-type Ca2+ channel current, I CaL, for different depolarizing potentials in the range −40 mV to 0 mV at voltage steps of 10 mV with a holding potential V h = −60mV, shown both as a function of (a) time and (b) depolarizing potential V d, superimposed with results obtained using the model of Tong et al. [41]. (c) Behaviour of T-type Ca2+ channel current, I CaT, for different depolarizing potentials in the range −60 mV to 20 mV, with a holding potential V h = −80mV. (TIFF) [file pone.0118443.s002.tiff]

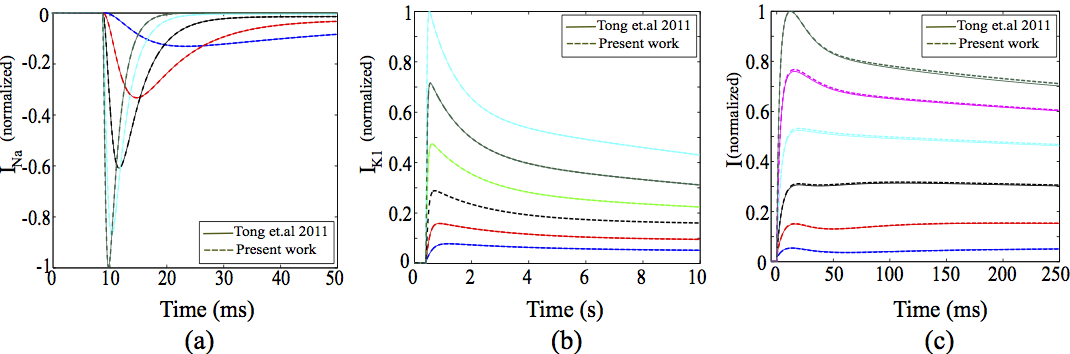

Supplement: S2 Fig — (a) Behaviour of Na+ channel current, I Na, at different depolarizing potentials in the range −40 mV to 20 mV with a holding potential V h = −40 mV. (b) Behaviour of the K+ channel current I K1 for g k = 0.8nS/pF, at different depolarizing potentials in the range −40 mV to 10 mV with a holding potential V h = −80 mV, normalized to the peak current at 10 mV. (c) Behaviour of the total K+ channel current, at different depolarizing potentials in the range −30 mV to 70 mV with a holding potential V h = −80 mV, normalized to the peak current at V d = 70 mV. (TIFF) [file pone.0118443.s003.tiff]

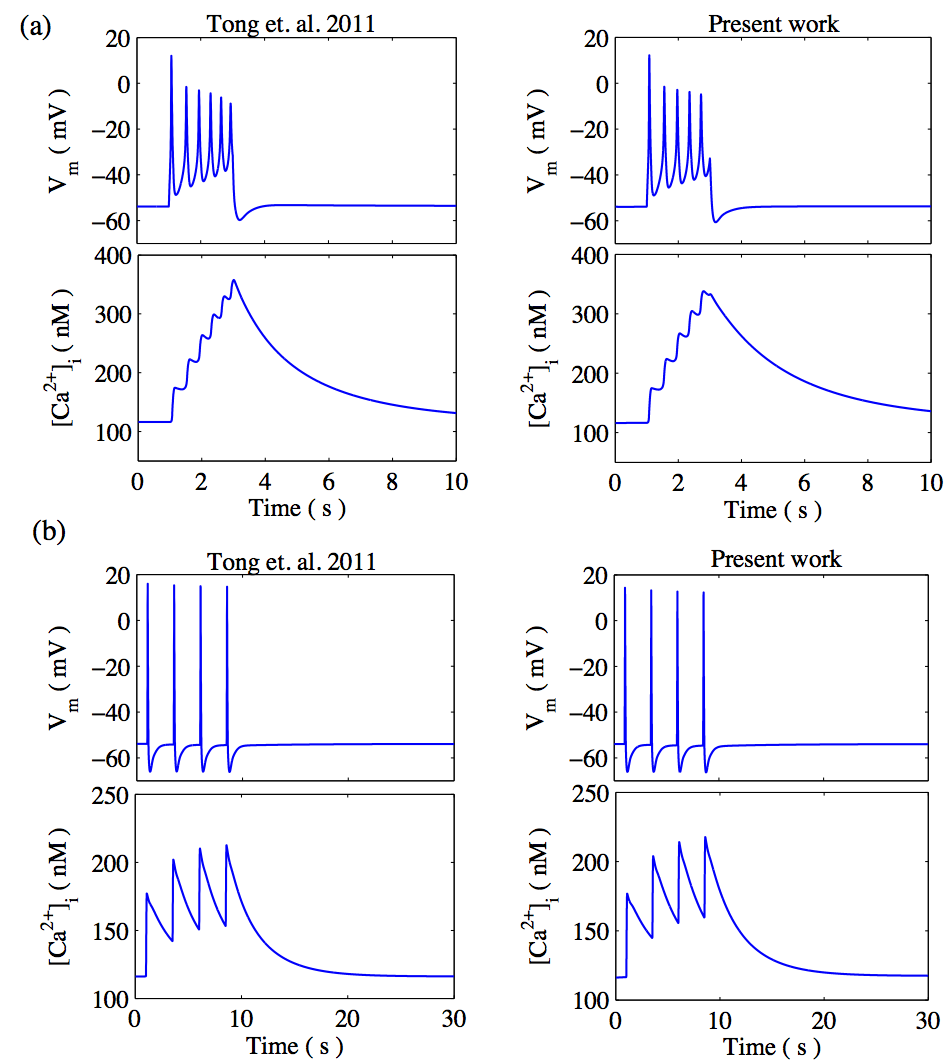

Supplement: S3 Fig — Action potentials using the model used in this article, compared with the corresponding results obtained using the model of Tong et al. [41], for the situations where: (a) A depolarizing current clamp of amplitude I st = −0.5pA/pF is applied for two seconds under control conditions (c.f. Figure 12 of Tong et al. [41]). (b) A stimulus of amplitude −1.5 pA/pF is applied over 20 ms at 0.4 Hz (c.f. Figure 13 of Tong et al. [41]). (TIFF) [file pone.0118443.s004.tiff]

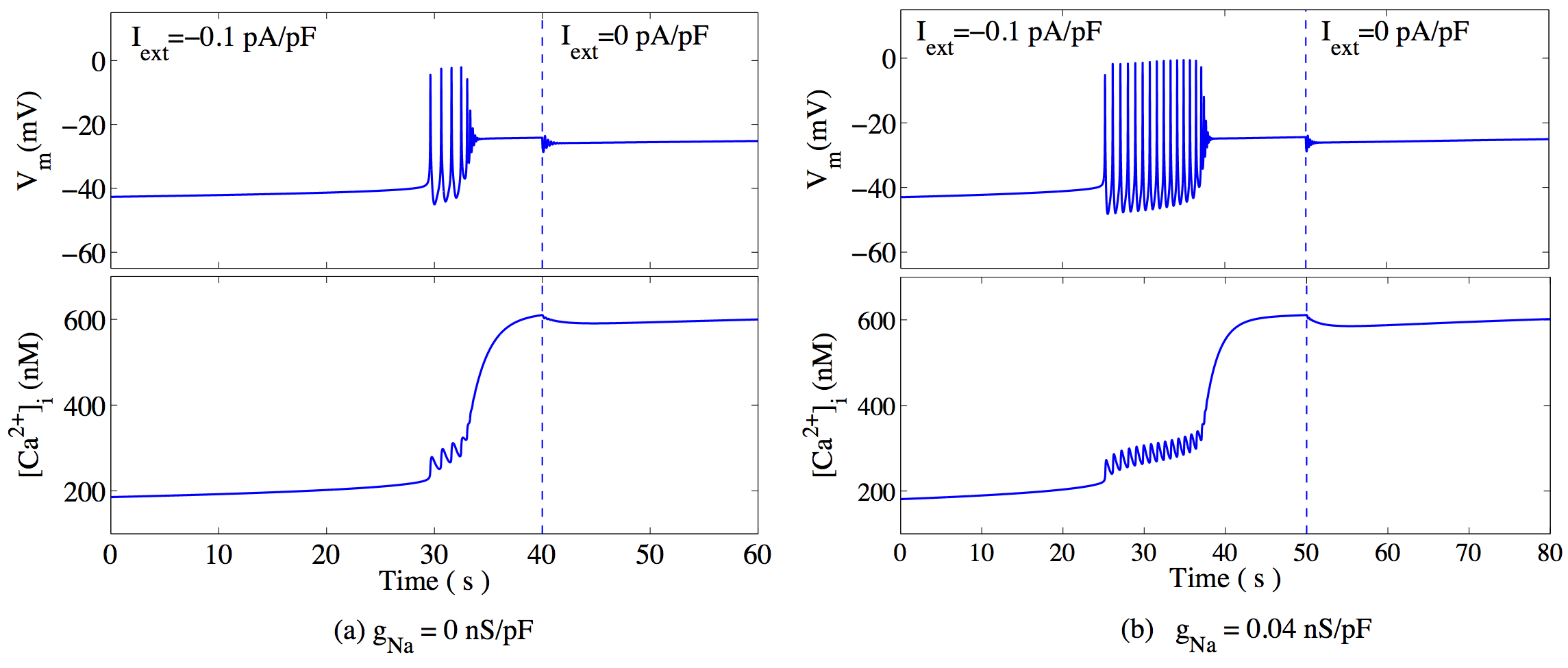

Supplement: S4 Fig — Behaviour of the model of Tong et al. [41], for the situation where a constant stimulus (I st = −0.1 pA/pF) is applied for two values of the sodium conductance, viz. (a) g Na = 0 nS/pF, and (b) g Na = 0.04 nS/pF. The evolution of the [top] membrane potential, and [bottom] intracellular calcium concentration is displayed in each case. The vertical dashed line indicates the time at which the stimulus is turned off. (TIFF) [file pone.0118443.s005.tiff]

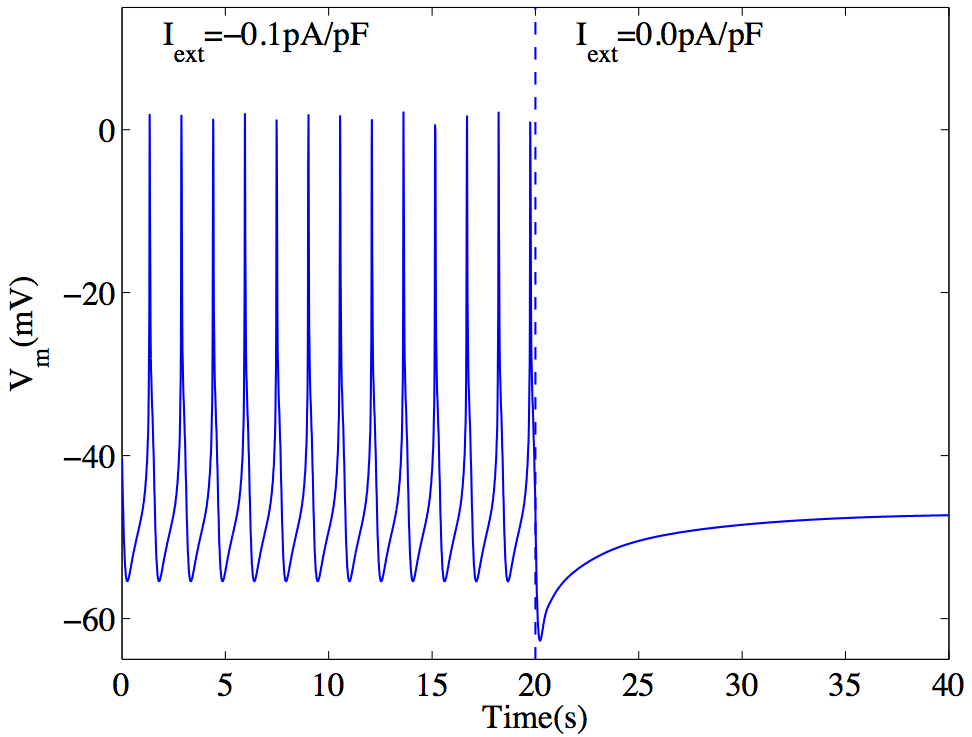

Supplement: S5 Fig — Behaviour of our model for the situation where a constant stimulus (I st = -0.1pA/pF) is applied. When the current is turned off (at the time indicated by vertical dashed line), the oscillations cease, and the system eventually returns to its resting state. (TIFF) [file pone.0118443.s006.tiff]

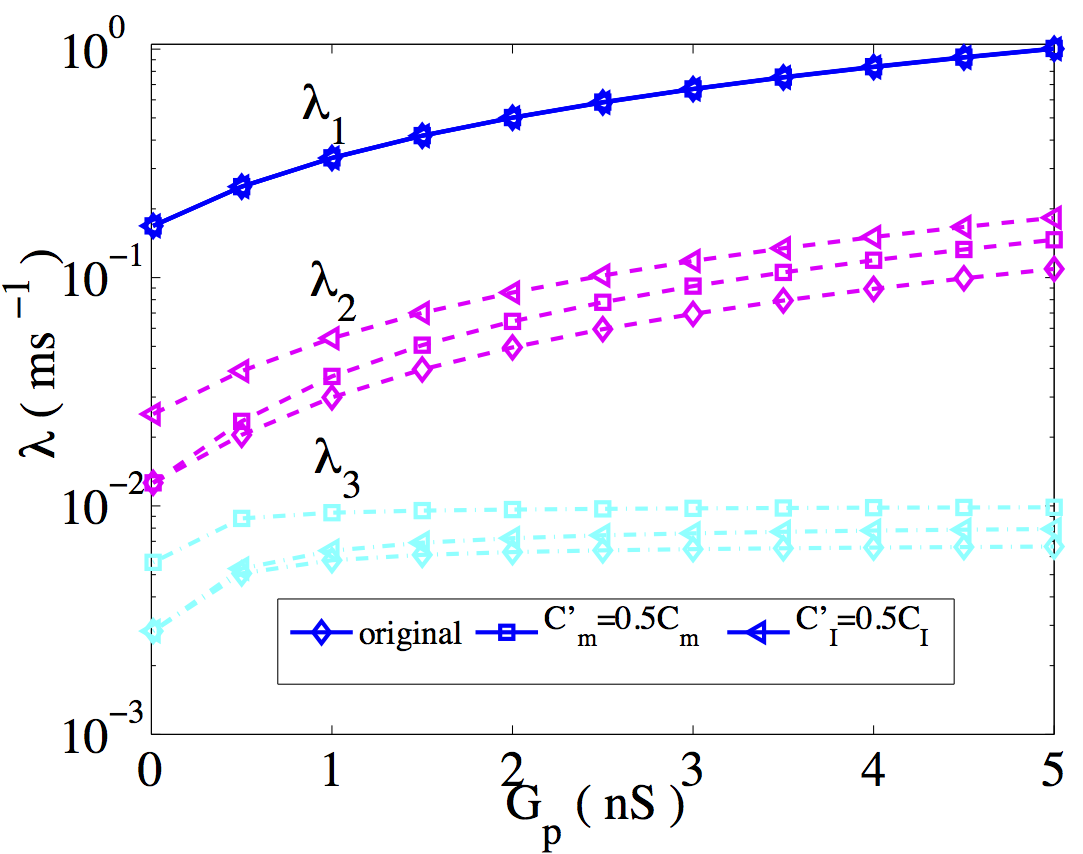

Supplement: S6 Fig — Decay rates λ 1,2,3 of membrane potentials V m, V I and V F, respectively. When C m (C I) is changed by 50%, while leaving C F and C I (C m) unchanged, it can be seen that the fibroblast has the largest decay rate that is one order of magnitude larger than the others. The method is applied to determine the decay rate of the other cells. (TIFF) [file pone.0118443.s007.tiff]

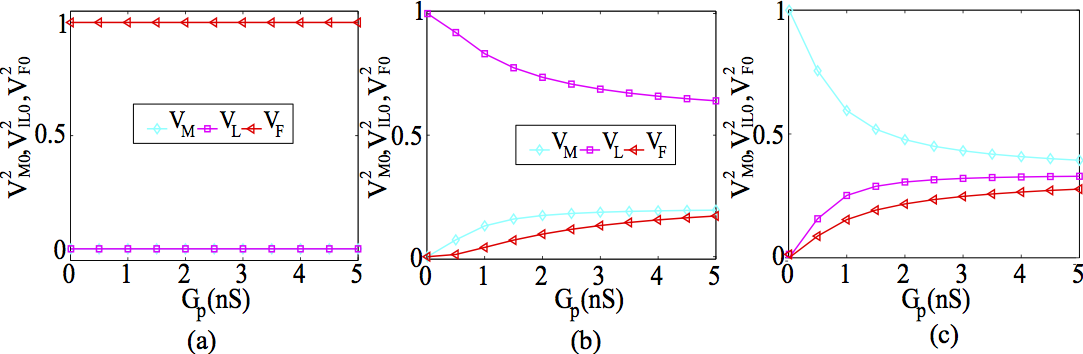

Supplement: S7 Fig — Components of eigenvectors associated with (a) λ 1, (b) λ 2 and (c) λ 3. (TIFF) [file pone.0118443.s008.tiff]

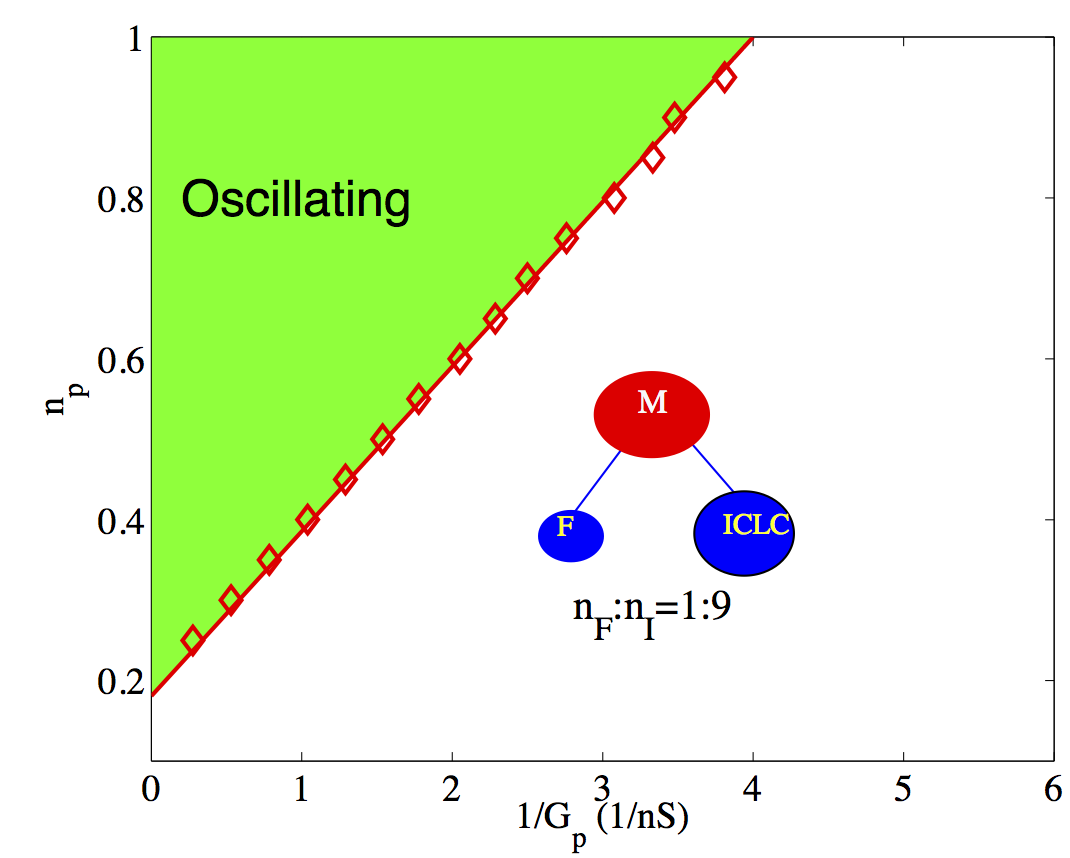

Supplement: S8 Fig — The (n p, G p) parameter space for the case of a single myocyte coupled to n I ICLCs and n F fibroblasts, indicating the region where oscillations are observed. The ratio of fibroblasts to ICLCs, nF:nI is 1:9. For comparison with the results of coupling a myocyte with an effective passive cell see Fig. 4 in the main text. (TIFF) [file pone.0118443.s009.tiff]
